# Analysis of adverse drug reactions of Denosumab (Prolia) in osteoporosis based on FDA adverse event reporting system (FAERS)

**DOI:** 10.3389/fphar.2024.1358592

**Published:** 2024-06-10

**Authors:** Ruibo Li, Xingyue Yuan, Xi Chen, Yili Ou, Jin Chen

**Affiliations:** ^1^ Department of Orthopaedics, Deyang Peoples’ Hospital, Deyang, Sichuan, China; ^2^ Department of Pathology, Deyang Peoples’ Hospital, Deyang, Sichuan, China

**Keywords:** osteoporosis, Denosumab, adverse drug reactions, pharmacovigilance, FDA adverse event reporting system

## Abstract

**Objective:**

To comprehensively analyze the ADRs associated with Denosumab (Prolia) in the treatment of osteoporosis using data from the FAERS database, and gain a better understanding of the potential risks and side effects of Denosumab (Prolia) therapy.

**Methods:**

Data of Denosumab (Prolia) were collected from the FAERS database covering the period from first quarter of 2010 to the third quarter of 2023. Disproportionality analysis was performed by calculating the reporting odds ratios (ROR), proportional reporting ratio (PRR), and Bayesian analysis confidence propagation neural network (BCPNN) to detect positive signals.

**Results:**

Totally, 17,985,365 reports were collected from the FAERS database, 1,97,807 reports of Denosumab (Prolia) were identified as the “primary suspected (PS)” ADRs. Denosumab (Prolia) induced ADRs occurred in 27 organ systems. 38 significant disproportionality PTs satisfying with the three algorithms were retained at the same time. Unexpected significant ADRs such as bone density abnormal and immobile also occur. The majority of the ADRs occurred within the first 30 days after Denosumab (Prolia) initiation.

**Conclusion:**

Based on the American FAERS database, the high frequency ADRs of Denosumab (Prolia) were hypocalcaemia, bone density abnormal, eczema, rebound effect, spinal deformity, etc. Clinical use of this drug should focus on this part of ADRs. Attention should also be paid to newly discovered ADRs, such as immobile, menopausal symptoms, etc., to avoid more serious consequences. Cohort studies, more detailed and comprehensive case information, and long-term clinical investigations are needed to confirm these results and to further understand the safety profile of Denosumab (Prolia).

## 1 Introduction

Osteoporosis is a chronic skeletal disorder characterized by reduced bone density and increased risk of fractures. It affects millions of individuals worldwide, particularly postmenopausal women and the elderly population. In 2017–2018, the age-adjusted prevalence of osteoporosis in adults 50 years and older in the United States was 12.6% (Sarafrazi et al., 2021). In China, among people aged 40 and above, 5.0 percent of men suffer from osteoporosis, while the prevalence of osteoporosis among women reaches a staggering 20.6 percent (Wang et al., 2021). The clinical and economic burden of osteoporosis-related fractures is substantial. Osteoporosis accounts for greater than 90% of hip and vertebral fractures in women aged 65–84 years (NIH Consensus Development Panel, 2001). In the United States alone, osteoporosis is responsible for over two million broken bones each year, resulting in healthcare costs exceeding $19 billion annually. By 2025, these costs are projected to rise to approximately $25.3 billion (Center and Bliuc, 2021).

Currently, lifestyle recommendations (vitamin D and calcium supplementation, exercise, and smoking alcohol cessation) and antiresorptive agents as standard therapies for osteoporosis, with bisphosphonates as first-line treatment, were proved to have beneficial effects on bone mineral density (BMD) and risk of fragile fractures in postmenopausal women (Cosman et al., 2014; Qaseem et al., 2017). However, for some patients, these treatments may not be effective. For better and more effective management of osteoporosis, Denosumab, a monoclonal antibody targeting a nuclear factor-κB ligand receptor activator (RANKL), was developed (Pang et al., 2020).

Denosumab has two different drug specifications. Xgeva (120 mg) is used for preventing bone-related events in cancer patients, and Prolia (60 mg) is used for treating osteoporosis. Denosumab (Prolia) has emerged as a promising therapeutic intervention for osteoporosis due to its ability to inhibit bone resorption by decreasing RANKL-induced osteoclast activity (Hanley et al., 2012). It has been approved by regulatory agencies, such as the US Food and Drug Administration (FDA), as a treatment option for postmenopausal women with osteoporosis at high risk of fracture, as well as for men with osteoporosis. Randomized controlled studies and meta-analyses have shown that, compared with bisphosphonates, Denosumab (Prolia) not only significantly improves bone mineral density and reduces the incidence of osteoporotic fractures, but also does not significantly increase the occurrence of adverse events (Miller et al., 2016; Lyu et al., 2019).

Due to the limitations of clinical trials, some delayed and rare adverse events (AEs) may remain undetected, and safety information can be supplemented through post-market data analysis. The FDA Adverse Event Reporting System (FAERS) is a powerful tool that allows for the analysis of adverse events associated with drug use. It captures reports of adverse events submitted by healthcare professionals, patients, and manufacturers, providing valuable insights into the safety profile of medications (Sakaeda et al., 2013; Woo, 2014). Although some researchers have analyzed the adverse reactions of Denosumab based on FAERS and compared them with the adverse reactions of zoledronic acid, they have not conducted a comprehensive and detailed analysis of the possible adverse drug reactions (ADRs) that may occur during the treatment of osteoporosis with Denosumab (Prolia) (Oteo-Álvaro et al., 2023; Su et al., 2023).

This study aims to comprehensively analyze the ADRs associated with Denosumab (Prolia) in the treatment of osteoporosis using data from the FAERS database. By examining the reported ADRs, we can gain a better understanding of the potential risks and side effects of Denosumab (Prolia) therapy.

The findings from this analysis will provide insights into the safety profile of Denosumab (Prolia), aiding healthcare professionals in making informed decisions regarding treatment choices for patients with osteoporosis. Furthermore, it may guide future research and development efforts to enhance the safety and efficacy of Denosumab (Prolia), ultimately improving patient outcomes.

Overall, this study contributes to the ongoing efforts to optimize pharmacovigilance and promote patient safety by utilizing the vast information available in the FAERS database to assess the ADRs associated with Denosumab (Prolia) in the context of osteoporosis management.

## 2 Materials and methods

### 2.1 Data source and collection

The data in this study were derived from the FAERS database in the United States. The FAERS database, which has been freely available since 2004, collects post-marketing ADRs and is updated quarterly. Seven databases make up FAERS data files, including demographic and administrative information (DEMO), adverse drug reaction information (REAC), patient outcome information (OUTC), drug information (DRUG), drug therapy starts dates and end dates (THER), information on report sources (RPSR), and indications for use/diagnosis (INDI). ADRs were collected from the first quarter of 2010 to the third quarter of 2023 based on the launch date of Denosumab (Prolia).

### 2.2 Data processing

**FIGURE 1:**
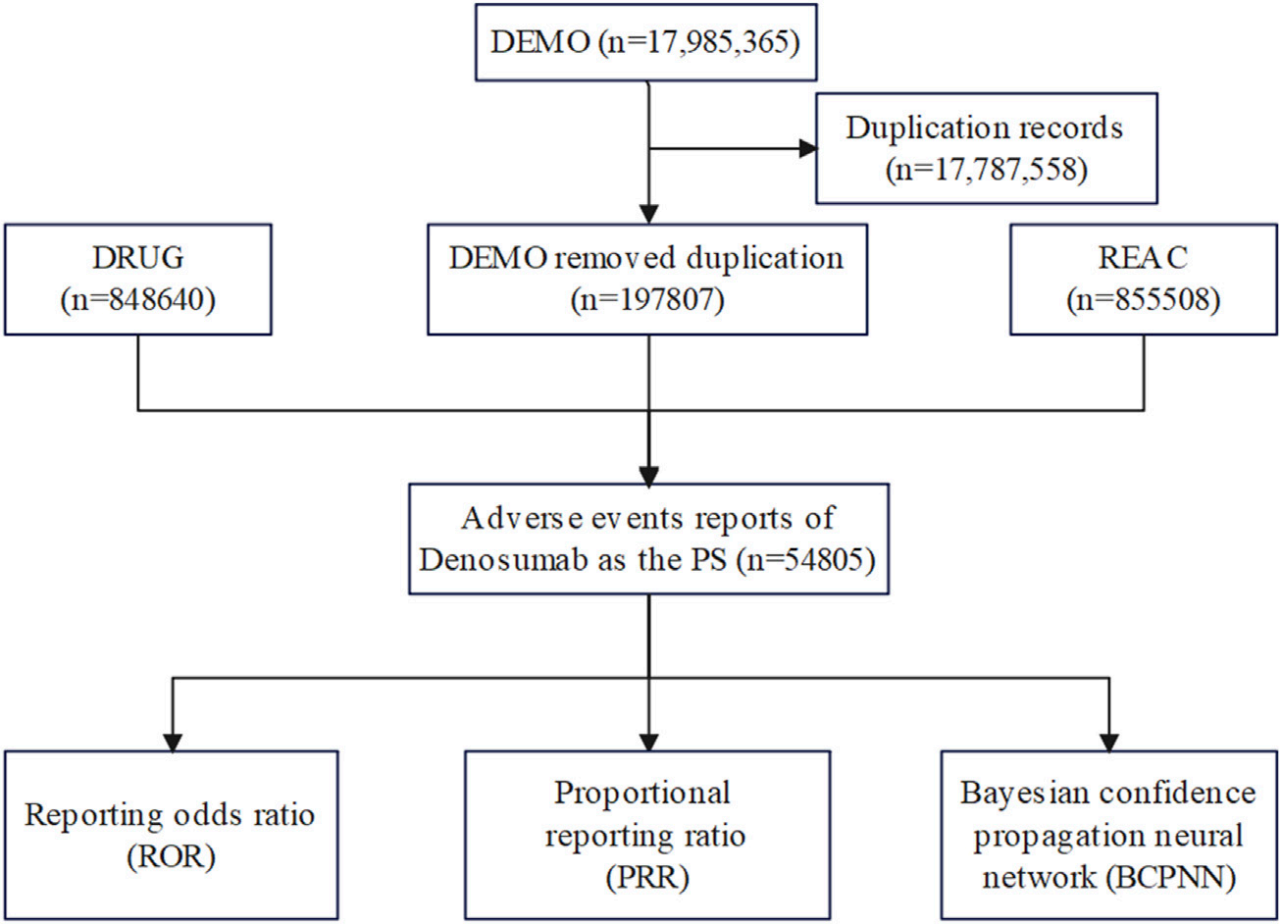
Flow diagram of the study (DEMO, demographic and administrative information; DRUG drug Information; REAC, preferred terminology for adverse drug reactions; PS, primary suspect drug).

Write the downloaded XML data package into RStudio and clean the data following the recommendations from the FDA. This process involved extracting relevant fields that pertain to our study, particularly focusing on demographic and drug information (DEMO and DRUG files). Perform a query using the generic name “Denosumab (Prolia)" and the trade name “Prolia” as targe drugs, “osteoporosis” as indication, and include only ADRs reports where Denosumab (Prolia) is the primary suspected drug (PS). A major problem in spontaneous reporting data is the presence of duplicates (i.e., the same report submitted by different sources) and multiple reports (i.e., a follow-up of the same case with additional and updated information). In the present study a two-step procedure of deduplication was applied. Firstly, only the last version of cases for which a follow-up was available was used. Secondly, cases with the same event, event date, age, gender, and country of origin were considered as duplicated (Cirmi et al., 2020). For ADRs names in the reports, use the preferred term (PT) from the Medical Dictionary for Regulatory Activities (MedDRA) for standardized encoding. The cleaned and standardized data was then compiled into a final dataset, ready for analysis. This dataset included only those reports where Denosumab (Prolia) was listed as the primary suspected drug (PS), aligning with our study’s focus. During the study period, 17,985,365 reports related to Denosumab (Prolia) were obtained from the FAERS database. After excluding duplicates, 1,97,807 reports identified Denosumab (Prolia) as the PS, and 54,805 ADRSs were associated with Denosumab (Prolia) (Figure 1). All ADRs reports for Denosumab (Prolia) were analyzed at the System Organ Class (SOC) and PT levels.

### 2.3 Statistical analysis

The association between Denosumab (Prolia) and adverse reactions was determined using the Reporting Odds Ratio (ROR), Proportional Reporting Ratio (PRR), and Bayesian Confidence Propagation Neural Network (BCPNN) algorithms (Caldito et al., 2021), while excluding ADRs unrelated to medication use. The equations and criteria for the three algorithms are described in Table 1. ADRs signals that satisfied all three algorithm criteria were considered significant signals. Significant signals not listed in the package insert were considered new signals. Additionally, the onset time was defined as the date of initiation of drug use to the time of occurrence of adverse reactions (Yin et al., 2022).

**TABLE 1:** There major algorithms used for signal detection.

Algorithms	Equation	Criteria
ROR	ROR = ad/b/c	lower limit of 95% CI > 1, N≥3
	95%CI = e^ln(ROR)±1.96(1/a+1/b+1/c+1/d)^0.5^	
PRR	PRR = a(c + d)/c/(a+b)	PRR≥2, χ^2^≥4, N≥3
	χ^2^ = [(ad-bc)^2](a+b + c + d)/[(a+b) (c + d) (a+c) (b + d)]	
BCPNN	IC = log_2_a(a+b + c + d) (a+c) (a+b)	IC025 > 0
	95%CI = E(IC) ± 2V(IC)^0.5	
	95%CI = e^ln(EBGM)±1.96(1/a+1/b+1/c+1/d)^0.5^	

Equation: a, number of reports containing both the target drug and target adverse drug reaction; b, number of reports containing other adverse drug reaction of the target drug; c, number of reports containing the target adverse drug reaction of other drugs; d, number of reports containing other drugs and other adverse drug reactions. 95%CI, 95% confidence interval; *N*, the number of reports; χ^2^, chi-squared; IC, information component; IC025, the lower limit of 95% CI, of the IC; E(IC), the IC, expectations; V(IC), the variance of IC.

Data processing was carried out using Microsoft Excel 2023 and RStudio (Version 4.3.1.).

## 3 Results

### 3.1 General characteristics

Clinical characteristics of ADRs to Denosumab (Prolia) are shown in Table 2. In terms of gender, approximately 90.50% were female and 6.04% were male. Regarding age, the age group of 65–85 years accounted for the highest proportion at approximately 47.94%, followed by the age group of 18–64 years (15.44%), the age group over 85 years (9.65%), and the age group under 18 years (0.03%).

**TABLE 2:** Clinical characteristics of ADRs to Denosumab (Prolia).

Characteristics	Case number, n	Case proportion, %
Number of events	54,805	
Gender		
Male	3,312	6.04
Female	49,599	90.50
Missing	1894	3.46
Age (year)		
<18	17	0.03
18–64	8,460	15.44
65–85	26,276	47.94
>85	5,290	9.65
Missing	14,762	26.94
Reported Countries (Top five)		
America	39,785	72.59
Canada	3,907	7.13
Japan	1815	3.31
United Kingdom	1,149	2.10
Germany	1,044	1.90
Reported Person		
Physician	26,414	48.20
Consumer	17,104	31.21
Other health-professional	6,637	12.11
Health professional	2,271	4.14
Pharmacist	1771	3.23
Registered Nurse	7	0.01
Lawyer	4	0.01
Missing	597	1.09
Reporting year		
2010	78	0.14
2011	459	0.84
2012	2,707	4.94
2013	3,804	6.94
2014	5,649	10.31
2015	3,981	7.26
2016	5,415	9.88
2017	9,994	18.24
2018	8,734	15.94
2019	4,154	7.58
2020	3,194	5.83
2021	2,924	5.34
2022	2,196	4.01
2023	1,516	2.77

ADRs, adverse drug reactions.

The top five reporting countries were America (72.95%), Canada (7.13%), Japan (3.31%), United Kingdom (2.10%), and Germany (1.90%). Excluding unknown reporters, physicians reported the highest number of adverse events at 48.20%, followed by consumers at 31.21%.

The year with the highest number of ADRs reports was 2017 at 18.24%, followed by 2018 (15.94%), 2014 (10.31%), 2016 (9.88%), and 2019 (7.58%).

### 3.2 Signal detection

The signal strength of Denosumab (Prolia) at the System Organ Class (SOC) level is reported in Table 3. Based on the analysis, we identified 27 organ systems involved in ADRs induced by Denosumab (Prolia).

**TABLE 3:** The signal strength of Denosumab (Prolia) at the System Organ Class (SOC) level.

System organ class (SOC)	Denosumab (Prolia) cases reporting SOC	ROR (95% two-sided CI)	PRR (X^2^)	IC (IC025)
Musculoskeletal and connective tissue disorders	29,269	1.32 (1.30–1.34)	1.26 (1,460.08)	0.27 (−1.40)
Injury, poisoning and procedural complications	22,488	1.32 (1.30–1.34)	1.27 (1,185.06)	0.28 (−1.38)
General disorders and administration site conditions	21,689	0.98 (0.96–0.99)	0.98 (7.00)	−0.02 (−1.69)
Gastrointestinal disorders	12,279	0.88 (0.86–0.90)	0.89 (150.68)	−0.14 (−1.80)
Skin and subcutaneous tissue disorders	9,598	2.18 (2.12–2.23)	2.10 (3,991.41)	0.82 (−0.85)
Nervous system disorders	8,051	0.69 (0.67–0.71)	0.71 (915.53)	−0.42 (−2.09)
Infections and infestations	7,848	1.05 (1.02–1.07)	1.04 (12.05)	0.05 (−1.62)
Investigations	6,827	0.86 (0.84–0.88)	0.86 (129.94)	−0.18 (−1.84)
Respiratory, thoracic and mediastinal disorders	3,968	0.71 (0.68–0.73)	0.71 (412.25)	−0.41 (−2.08)
Surgical and medical procedures	3,953	1.02 (0.98–1.05)	1.02 (0.86)	0.02 (−1.65)
Psychiatric disorders	3,076	0.56 (0.53–0.58)	0.56 (956.96)	−0.71 (−2.38)
Metabolism and nutrition disorders	2,860	0.81 (0.78–0.85)	0.82 (101.11)	−0.24 (−1.91)
Neoplasms benign, malignant and unspecified (incl cysts and polyps)	2,423	1.05 (1.01–1.10)	1.05 (5.44)	0.06 (−1.60)
Cardiac disorders	2,152	0.60 (0.58–0.63)	0.61 (488.86)	−0.61 (−2.28)
Vascular disorders	2086	0.64 (0.61–0.67)	0.64 (369.88)	−0.54 (−2.21)
Renal and urinary disorders	2050	0.78 (0.75–0.82)	0.79 (105.29)	−0.29 (−1.96)
Eye disorders	1,569	0.58 (0.55–0.61)	0.58 (430.34)	−0.68 (−2.34)
Ear and labyrinth disorders	1,137	1.14 (1.07–1.21)	1.14 (15.25)	0.15 (−1.51)
Immune system disorders	1,011	1.31 (1.22–1.40)	1.31 (57.29)	0.31 (−1.36)
Social circumstances	991	2.81 (2.60–3.04)	2.80 (723.86)	1.09 (−0.57)
Blood and lymphatic system disorders	683	0.42 (0.39–0.46)	0.43 (491.31)	−1.08 (−2.75)
Reproductive system and breast disorders	559	0.61 (0.56–0.67)	0.62 (119.33)	−0.60 (−2.27)
Endocrine disorders	529	0.89 (0.81–0.97)	0.89 (6.44)	−0.14 (−1.81)
Hepatobiliary disorders	421	0.52 (0.47–0.58)	0.52 (163.96)	−0.81 (−2.47)
Product issues	335	0.46 (0.41–0.51)	0.46 (198.98)	−0.99 (−2.66)
Congenital, familial and genetic disorders	85	0.50 (0.40–0.63)	0.51 (37.34)	−0.86 (−2.53)
Pregnancy, puerperium and perinatal conditions	7	0.21 (0.10–0.45)	0.21 (20.05)	−2.04 (−3.73)

ROR, reporting odds ratio; CI, confidence interval; PRR, proportional reporting ratio; X^2^, chi-squared, IC, information component; IC, 025, the lower limit of 95% CI, of the IC.

After excluding signals unrelated to drug treatment, such as product issues, various injuries, poisonings, procedure-related complications, surgeries, and medical operations, a total of 38 Preferred Terms (PTs) were identified as significant signals that met the criteria of all three algorithms (Table 4). The top 10 PTs with the highest number of reports are as follows: “Hypocalcaemia”, “Bone density abnormal”, “Eczema”, “Rebound effect”, “Spinal deformity”, “Rash papular”, “Immobile”, “Menopausal symptoms”, “Skin infection” and “Blood alkaline phosphatase decreased”. Interestingly, some of these adverse events were not documented in the drug label. These top 10 unexpected ADRs include “Bone density abnormal”, “Immobile”, “Menopausal symptoms”, “Blood alkaline phosphatase decreased”, “Hypogonadism”, “Root canal infection”, “Gingival cyst”, “Hungry bone syndrome”, “Stomatitis necrotising” and “Acute leukaemia” (Table 5). The top 10 new ADRs for Denosumab (Prolia) by association strength included “Gingival cyst”, “Immunoglobulins increased”, “Cutaneous sarcoidosis”, “Meningioma benign”, “Carbohydrate antigen 19–9 increased”, “Eyelid exfoliation”, “Oral neoplasm benign”, “HIV test positive”, “Paraganglion neoplasm” and “Respiratory tract oedema” (Table 6).

**TABLE 4:** Signal strength of reports of Denosumab (Prolia) at the Preferred Term (PT) level in FAERS database.

System organ class (SOC)	Preferred terms (PTs)	Denosumab (Prolia) cases reporting PT	ROR (95% two-sided CI)	PRR (X^2^)	IC (IC025)
Social circumstances	Immobile	89	10.14 (7.03–14.64)	10.13 (234.97)	1.97 (0.29)
	housebound	3	14.35 (1.49–137.94)	14.35 (9.31)	2.12 (0.01)
Skin and subcutaneous tissue disorders	Eczema	347	6.68 (5.67–7.86)	6.66 (698.53)	1.75 (0.08)
	Rash papular	131	7.94 (6.00–10.49)	7.93 (298.56)	1.85 (0.17)
	Macule	39	16.96 (8.69–33.12)	16.96 (128.84)	2.17 (0.46)
	Rash vesicular	26	6.55 (3.62–11.83)	6.54 (51.57)	1.74 (0.02)
	Eczema nummular	8	9.57 (2.88–31.77)	9.57 (20.45)	1.95 (0.10)
	Cutaneous sarcoidosis	4	19.13 (2.14–171.17)	19.13 (13.75)	2.21 (0.17)
Respiratory, thoracic and mediastinal disorders	Respiratory tract oedema	3	14.35 (1.49–137.94)	14.35 (9.31)	2.12 (0.01)
Reproductive system and breast disorders	Menopausal symptoms	77	10.23 (6.89–15.20)	10.23 (204.30)	1.98 (0.29)
Neoplasms benign, malignant and unspecified (incl cysts and polyps)	Acute leukaemia	5	11.96 (2.32–61.63)	11.96 (14.34)	2.05 (0.09)
	Meningioma benign	4	19.13 (2.14–171.17)	19.13 (13.75)	2.21 (0.17)
	Oral neoplasm benign	4	19.13 (2.14–171.17)	19.13 (13.75)	2.21 (0.17)
	Paraganglion neoplasm	3	14.35 (1.49–137.94)	14.35 (9.31)	2.12 (0.01)
	Leiomyosarcoma	3	14.35 (1.49–137.94)	14.35 (9.31)	2.12 (0.01)
Musculoskeletal and connective tissue disorders	Spinal deformity	175	5.90 (4.73–7.36)	5.89 (318.68)	1.67 (0.00)
	Hungry bone syndrome	6	9.57 (2.39–38.25)	9.57 (15.34)	1.97 (0.05)
	Muscle discomfort	4	19.13 (2.14–171.17)	19.13 (13.75)	2.21 (0.17)
Metabolism and nutrition disorders	Hypocalcaemia	763	8.56 (7.63–9.67)	8.55 (1826.77)	1.89 (0.22)
Investigations	Bone density abnormal	381	8.42 (7.12–9.94)	8.40 (901.54)	1.88 (0.21)
	Blood alkaline phosphatase decreased	41	6.54 (4.08–10.47)	6.54 (81.24)	1.74 (0.04)
	Investigation	31	8.72 (4.83–15.76)	8.72 (75.06)	1.90 (0.18)
	Calcium ionized decreased	6	14.35 (2.90–71.09)	14.35 (18.63)	2.12 (0.19)
	Immunoglobulins increased	4	19.13 (2.14–171.17)	19.13 (13.75)	2.21 (0.17)
	Carbohydrate antigen 19–9 Increased	4	19.13 (2.14–171.17)	19.13 (13.75)	2.21 (0.17)
	HIV test positive	3	14.35 (1.49–137.94)	14.35 (9.31)	2.12 (0.01)
Infections and infestations	Skin infection	74	6.21 (4.40–8.77)	6.21 (140.73)	1.71 (0.02)
	Root canal infection	16	12.75 (4.99–32.60)	12.75 (47.27)	2.07 (0.30)
	Ear infection fungal	5	23.91 (2.79–204.70)	23.91 (18.30)	2.27 (0.28)
	Viral myocarditis	3	14.35 (1.49–137.94)	14.35 (9.31)	2.12 (0.01)
	Streptococcal urinary tract Infection	3	14.35 (1.49–137.94)	14.35 (9.31)	2.12 (0.01)
	Acne pustular	3	14.35 (1.49–137.94)	14.35 (9.31)	2.12 (0.01)
General disorders and administration site conditions	Rebound effect	280	20.02 (15.34–26.14)	19.99 (975.55)	2.22 (0.55)
Gastrointestinal disorders	Gingival cyst	9	21.52 (4.65–99.62)	21.52 (32.02)	2.24 (0.38)
	Stomatitis necrotizing	5	11.97 (2.32–61.63)	11.96 (14.34)	2.05 (0.09)
	Tooth pulp haemorrhage	3	14.35 (1.49–137.94)	14.35 (9.31)	2.12 (0.01)
Eye disorders	Eyelid exfoliation	4	19.13 (2.14–171.17)	19.13 (13.75)	2.21 (0.17)
Endocrine disorders	Hypogonadism	23	6.47 (3.46–12.11)	6.47 (45.21)	1.73 (0.01)

ROR, reporting odds ratio; CI, confidence interval; PRR, proportional reporting ratio; X^2^, chi-squared, IC, information component; IC, 025, the lower limit of 95% CI, of the IC.

**TABLE 5:** The top 10 new ADRs of Denosumab (Prolia) by frequency of reporting.

Preferred terms (PTs)	Denosumab (Prolia) cases reporting PT	ROR (95% two-sided CI)	PRR (X^2^)	IC (IC025)
Bone density abnormal	381	8.42 (7.12–9.94)	8.40 (901.54)	1.88 (0.21)
Immobile	89	10.14 (7.03–14.64)	10.13 (234.97)	1.97 (0.29)
Menopausal symptoms	77	10.23 (6.89–15.20)	10.23 (204.30)	1.98 (0.29)
Blood alkaline phosphatase decreased	41	6.54 (4.08–10.47)	6.54 (81.24)	1.74 (0.04)
Hypogonadism	23	6.47 (3.46–12.11)	6.47 (45.21)	1.73 (0.01)
Root canal infection	16	12.75 (4.99–32.60)	12.75 (47.27)	2.07 (0.30)
Gingival cyst	9	21.52 (4.65–99.62)	21.52 (32.02)	2.24 (0.38)
Hungry bone syndrome	6	9.57 (2.39–38.25)	9.57 (15.34)	1.97 (0.05)
Stomatitis necrotising	5	11.97 (2.32–61.63)	11.96 (14.34)	2.05 (0.09)
Acute leukaemia	5	11.96 (2.32–61.63)	11.96 (14.34)	2.05 (0.09)

ADRs, adverse drug reactions; ROR, reporting odds ratio; CI, confidence interval; PRR, proportional reporting ratio; X^2^, chi-squared, IC, information component; IC, 025, the lower limit of 95% CI, of the IC.

**TABLE 6:** The top 10 new ADRs of Denosumab (Prolia) by association strength.

Preferred terms (PTs)	Denosumab (Prolia) cases reporting PT	ROR (95% two-sided CI)	PRR (X^2^)	IC (IC025)
Gingival cyst	9	21.52 (4.65–99.62)	21.52 (32.02)	2.24 (0.38)
Immunoglobulins increased	4	19.13 (2.14–171.17)	19.13 (13.75)	2.21 (0.17)
Cutaneous sarcoidosis	4	19.13 (2.14–171.17)	19.13 (13.75)	2.21 (0.17)
Meningioma benign	4	19.13 (2.14–171.17)	19.13 (13.75)	2.21 (0.17)
Carbohydrate antigen 19–9 increased	4	19.13 (2.14–171.17)	19.13 (13.75)	2.21 (0.17)
Eyelid exfoliation	4	19.13 (2.14–171.17)	19.13 (13.75)	2.21 (0.17)
Oral neoplasm benign	4	19.13 (2.14–171.17)	19.13 (13.75)	2.21 (0.17)
HIV test positive	3	14.35 (1.49–137.94)	14.35 (9.31)	2.12 (0.01)
Paraganglion neoplasm	3	14.35 (1.49–137.94)	14.35 (9.31)	2.12 (0.01)
Respiratory tract oedema	3	14.35 (1.49–137.94)	14.35 (9.31)	2.12 (0.01)

ADRs, adverse drug reactions; ROR, reporting odds ratio; CI, confidence interval; PRR, proportional reporting ratio; X^2^, chi-squared, IC, information component; IC, 025, the lower limit of 95% CI, of the IC.

### 3.3 Onset time of events

**FIGURE 2:**
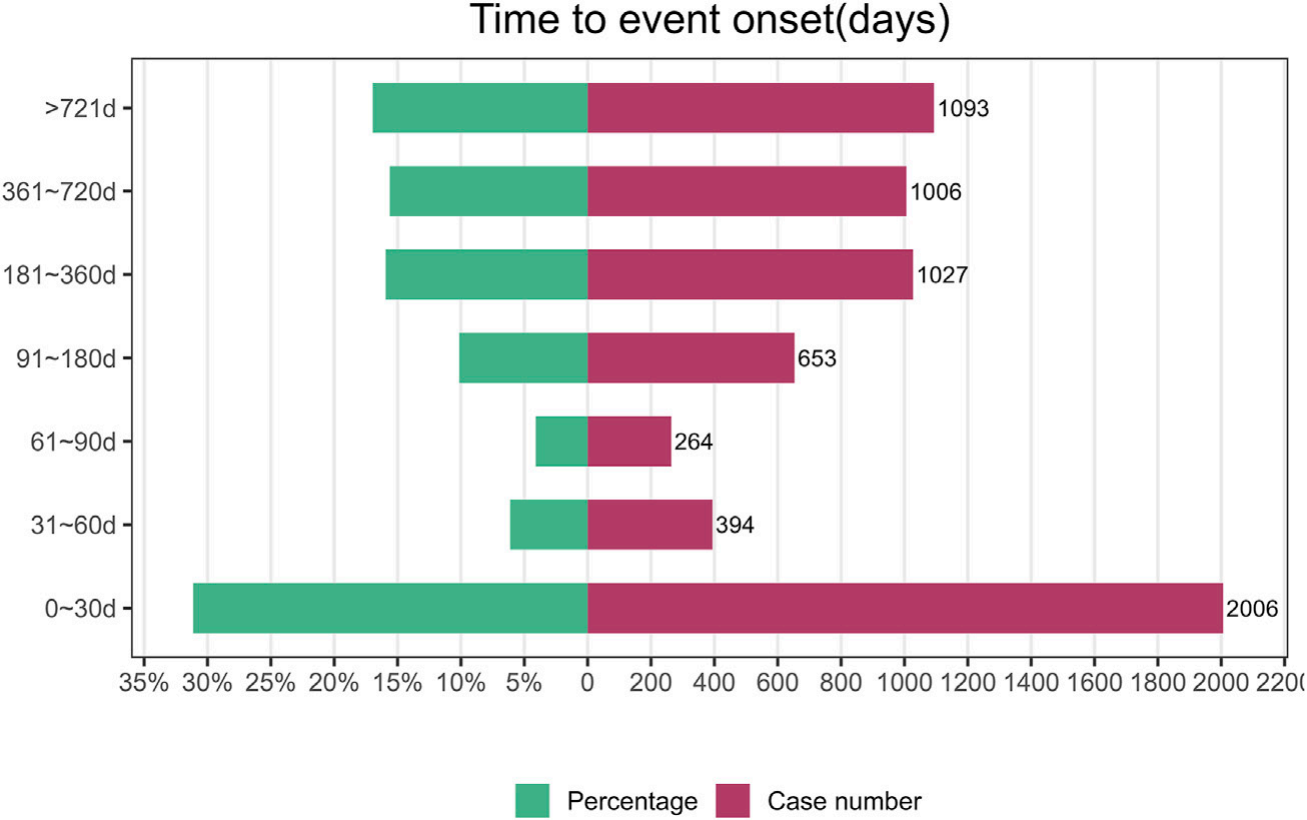
Time to onset of Denosumab (Prolia)-related ADRs (ADRs, adverse drug reactions).

The onset time of Denosumab (Prolia) associated AE was collected from the database. Excluding false positives, a total of 6,443 cases were reported. As shown in Figure 2, the majority of cases occurred within the first month after commencement of Denosumab (Prolia) use (n = 2006, 31.13%). Similar proportions of cases occurred after 6 months of treatment (n = 1,027, 15.94%), 1 year (n = 1006,15.61%), and 2 years (n = 1,093, 16.96%). ADRs occurred within 1–6 months of treatment in 20.36% of cases (n = 1,311).

## 4 Discussion

When searching for adverse event data related to Denosumab (Prolia) from the FAERS database, we focused on its indication for osteoporosis. In this study, we found that ADRs of Denosumab (Prolia) occurred more commonly in females (90.5%) than in males (6.04%). Additionally, the highest proportion of patients was in the age range of 65–85 years (47.94%). This aligns with the current epidemiological characteristics of osteoporosis (Compston et al., 2019) and indicates that the primary user population for Denosumab (Prolia) is elderly female osteoporosis patients. It is one of the most commonly prescribed antiresorptive drugs in clinical practice for the management of osteoporosis in postmenopausal women (Lewiecki and Bilezikian, 2012; Pang et al., 2020).

According to our study, among all the ADRs, hypocalcemia had the highest number of reported cases. However, the prescribing information for Denosumab (Prolia) lists hypocalcemia as “rare”. Based on the report by Cumming et al. (2009), the incidence of hypocalcemia in the treatment of osteoporosis with Denosumab (Prolia) is less than 0.05%. However, Huynh et al. (2016) reported that 14% of patients who received Denosumab (Prolia) in a tertiary hospital setting developed hypocalcemia within 6 months. This discrepancy might be attributed to under-reporting in clinical trials or differences in patient populations such as renal function status, vitamin D levels, and concomitant medications, which are not always fully accounted for in post-marketing surveillance data. The mechanism by which Denosumab induces hypocalcemia is primarily due to its potent inhibition of osteoclast-mediated bone resorption, leading to a rapid decrease in serum calcium levels. This effect, while beneficial for reducing bone turnover and increasing bone density, necessitates a balance in calcium homeostasis that may be disrupted, particularly in patients with existing risk factors (Daga and Joseph, 2021). Symptoms of hypocalcemia in clinical studies of Denosumab (Prolia) often include sensory abnormalities, muscle stiffness, muscle spasms, prolonged QT interval, etc. However, the majority of patients (59%) who developed Denosumab (Prolia)-associated hypocalcemia continued to receive Denosumab (Prolia) likely due to the lack of symptoms of hypocalcemia (Huynh et al., 2016).

Hypocalcemia, while clinically manageable, poses significant risks if not adequately monitored and treated. It is essential for healthcare providers to be aware of this risk, especially in populations with predisposing factors such as chronic kidney disease or those with severe vitamin D deficiency. We suggest that patients undergoing treatment with Denosumab (Prolia) should have their calcium and vitamin D levels closely monitored, and supplementation should be considered to mitigate the risk of hypocalcemia. In response to these findings, we recommend that clinical guidelines for the use of Denosumab (Prolia) in osteoporosis include more detailed advice on monitoring and managing calcium levels. Early identification of symptoms related to hypocalcemia and timely intervention can prevent serious complications associated with this condition.

According to the disproportionality analysis, the most significant signals at PT levels were ear infection fungal. Other ADRs classified as infections and infestations at the SOC level included viral myocarditis, streptococcal urinary tract infection, acne pustular, root canal infection, and skin infection. Among them, ear infection, streptococcal urinary tract infection, and skin infection were recorded in the drug package insert, and the others were newly discovered ADRs. A population-based cohort study found that, compared to the control group, patients treated with Denosumab (Prolia) for osteoporosis had a significantly higher risk of infectious diseases, including skin infections, urinary tract infections, acne pustular, fungal infections, etc. (Huang et al., 2023). The mechanism underlying the increased risk of infection with Denosumab (Prolia) is currently unclear but may be related to the critical role of RANKL expression in T cell function, which may promote T cell activation. In addition, the cellular immune response in osteoporosis patients receiving Denosumab (Prolia) may be inhibited to some extent (Walsh and Choi, 2014).

According to the disproportionality analysis, the most commonly reported signals at SOC levels were general disorders and administration site conditions. These ADRs included spinal deformity, hungry bone syndrome, and muscle discomfort. While Denosumab is indeed used to treat osteoporosis and reduces the risk of thoracolumbar osteoporotic fractures and spinal malformations, our study identified adverse events in some patients who reported abnormal bone density and spinal malformations following Denosumab treatment. We hypothesize that these adverse events may occur when osteoporosis patients experience further bone density loss during or after treatment, or when osteoporosis patients without spinal malformations before treatment develop spinal malformations during or after treatment. Hungry bone syndrome is a significant and rapid decrease in serum calcium levels that typically occurs following the surgical treatment of hyperparathyroidism but can occasionally be associated with osteoporosis treatment affecting bone metabolism. Symptoms might include muscle cramps, tingling, or cardiac complications due to hypocalcemia. Although hungry bone syndrome is not mentioned in the package insert of Denosumab (Prolia), previous literature reports suggest that it may present with symptoms resembling hungry bone syndrome during the treatment with Denosumab (Prolia) injection (Nachankar et al., 2022). As FAERS data is limited to adverse event reports and may not specify symptoms as part of a syndrome, individual symptoms like hypocalcemia are often recorded without the explicit label of hungry bone syndrome.

Due to the limitations of clinical trials, some late-onset and rare ADRs are difficult to detect, and the safety information of drugs can be supplemented by post-marketing data analysis. Through the disproportionality analysis, we found 24 kinds of unexpected ADRs not recorded in the drug instruction manual, of which the highest occurrence frequency was bone density abnormal (n = 381, ROR = 8.42). Gingival cyst (n = 9, ROR = 21.52) was the most strongly associated. The specific mechanism of bone density abnormal and gingival cyst in osteoporosis patients treated with Denosumab (Prolia) has not been reported in the literature, and further research is needed. Our results show that the emerging adverse events not recorded in the instruction manual have a high association strength and need to be paid enough attention to avoid causing serious consequences.

Furthermore, our findings indicate that the majority of cases occurred within 30 days following initiation of Denosumab (Prolia), with comparable rates of ADRs observed beyond 6 months of treatment duration. These results suggest the need for heightened vigilance regarding patient ADRs during the initial month, and early identification of Denosumab (Prolia)-induced ADRs could mitigate potential life-threatening consequences. In particular, the 1–6 months period in our study involved extensive aes experienced by patients shortly after beginning treatment with Denosumab (Prolia). This does not include the ‘Rebound’ effect, which is understood to occur after treatment is discontinued and can be determined by observation over a longer period of time (usually 1 year or more).

There are some limitations to this study: the FAERS database in the United States is a spontaneous reporting system, which often results in data loss such as missed reporting and incomplete case information, making it difficult to conduct a complete analysis, which may lead to certain biases in the results of this study. Meanwhile, the results of the measures of dis-proportionality method merely exhibit a statistical correlation and do not directly elucidate the causal relationship between drugs and ADRs. Therefore, further analysis of individual cases is imperative to facilitate a comprehensive evaluation of causality. However, despite the facts that FAERS database has some limitations in pharmacovigilance studies, this study still has significant implications for suggesting potential drug risks in clinical practice.

## 5 Conclusion

Based on the American FAERS database, the high frequency ADRs of Denosumab (Prolia) were hypocalcaemia, bone density abnormal, eczema, rebound effect, spinal deformity, etc. Clinical use of this drug should focus on this part of ADRs. Attention should also be paid to newly discovered ADRs, such as immobile, menopausal symptoms, etc., to avoid more serious consequences. Cohort studies, more detailed and comprehensive case information, and long-term clinical investigations are needed to confirm these results and to further understand the safety profile of Denosumab (Prolia).

## Data Availability

Publicly available datasets were analyzed in this study. This data can be found here: https://fis.fda.gov/sense/app/95239e26-e0be-42d9-a960-9a5f7f1c25ee/overview.
